# External Quality Assessment for Next-Generation Sequencing-Based HIV Drug Resistance Testing: Unique Requirements and Challenges

**DOI:** 10.3390/v12050550

**Published:** 2020-05-16

**Authors:** Emma R. Lee, Feng Gao, Paul Sandstrom, Hezhao Ji

**Affiliations:** 1 National HIV and Retrovirology Laboratories, National Microbiology Laboratory at JC Wilt Infectious Diseases Research Centre, Public Health Agency of Canada, Winnipeg, MB R3E 3R2, Canada; emmar.lee@canada.ca (E.R.L.); paul.sandstrom@canada.ca (P.S.); 2 Department of Medicine, Duke University Medical Center, Durham, NC 27710, USA; feng.gao@duke.edu; 3 Department of Medical Microbiology and Infectious Diseases, University of Manitoba, Winnipeg, MB R3E 0J9, Canada

**Keywords:** external quality assessment, next-generation sequencing, HIV, drug resistance testing, minority resistance variants

## Abstract

Over the past decade, there has been an increase in the adoption of next generation sequencing (NGS) technologies for HIV drug resistance (HIVDR) testing. NGS far outweighs conventional Sanger sequencing as it has much higher throughput, lower cost when samples are batched and, most importantly, significantly higher sensitivities for variants present at low frequencies, which may have significant clinical implications. Despite the advantages of NGS, Sanger sequencing remains the gold standard for HIVDR testing, largely due to the lack of standardization of NGS-based HIVDR testing. One important aspect of standardization includes external quality assessment (EQA) strategies and programs. Current EQA for Sanger-based HIVDR testing includes proficiency testing where samples are sent to labs and the performance of the lab conducting such assays is evaluated. The current methods for Sanger-based EQA may not apply to NGS-based tests because of the fundamental differences in their technologies and outputs. Sanger-based genotyping reports drug resistance mutations (DRMs) data as dichotomous, whereas NGS-based HIVDR genotyping also reports DRMs as numerical data (percent abundance). Here we present an overview of the need to develop EQA for NGS-based HIVDR testing and some unique challenges that may be encountered.

## 1. Introduction

Next-generation sequencing (NGS) or high throughput sequencing has revolutionized DNA sequencing methodology. This technology performs massive parallel sequencing of individual input templates which generates incredible amounts of data per run [1,2]. In comparison, conventional Sanger sequencing (SS) methods produce only a single consensus sequence per specimen, which represents the dominant genotypes in the test sample (Table 1). The main advantage of NGS over SS is its high sensitivity. In HIV drug resistance (HIVDR) genotyping, NGS could detect drug-resistance mutations (DRMs) present at frequencies below 20%, also known as minority resistance variants (MRVs), which are mostly undetectable by SS methods [1,3,4]. There is increasing evidence showing that the presence of MRVs may be clinically relevant since they are associated with an increase in the risk of treatment failure in patients who carry MRVs to the administered antiretroviral drugs [5,6,7,8,9,10,11]. As more labs are implementing NGS as the preferred platform for HIVDR genotyping, the need to develop appropriate external quality assessment (EQA) strategies suitable for such assays is urgent.

**Figure 1:**
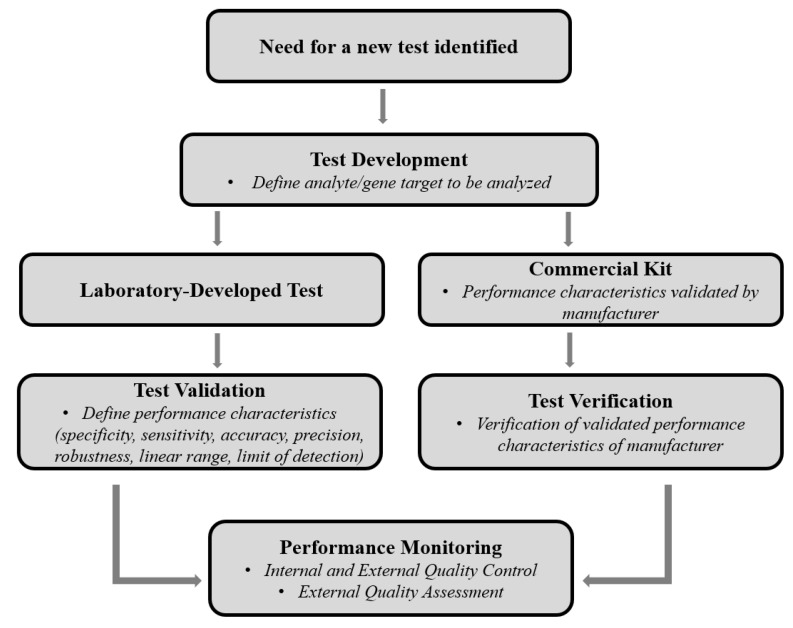
External quality assessment workflow for clinical tests (Adapted from https://euformatics.com/validation-for-ngs-based-clinical-tests/).

As with all other molecular clinical laboratory tests, NGS-based HIVDR assays must implement quality management systems to ensure high-quality test results [12,13,14,15,16]. When a lab starts a new clinical test, the test must be validated with quality assurance measures before the test can be administered for patient care purposes (Figure 1). Generally, a lab can use a commercial kit that has been approved by an appropriate regulatory agency (e.g., FDA), where performance specifications have been validated by the manufacturer. Alternatively, if no commercial options are available, the lab can independently develop its own test, specifically termed “Laboratory Developed Tests” (LDT), or sometimes referred to as “home-brew” or “in-house” tests. There are several considerations involved in LDTs, including target selection and data analysis approaches that are subjected to regulatory bodies such as Clinical and Laboratory Improvement Amendments (CLIA) and Clinical and Laboratory Standards Institute (CSLI) [17,18,19]. The assay must be validated by assessing multiple operational characteristics including accuracy, precision, analytical sensitivity, analytical specificity, robustness, reportable range and reference range [20]. Once the assay is validated and implemented, performance monitoring needs to be conducted regularly to ensure that the test continues to meet performance specifications. Performance monitoring includes internal and external quality controls, as well as participation in an EQA program [14,20].

EQA programs establish the comparison of assay performance amongst different participating labs, helping to identify analytical or interpretive errors, flag areas that need improvement, and identify training needs. EQA program uses methods such as proficiency testing (PT), re-testing, and on-site evaluation to check a lab’s assay performance conducted by a third party agency [16]. PT is the most commonly applied EQA approach for biomedical assays such as HIVDR testing. With a PT-oriented EQA program, participant labs receive blind-coded samples, process the samples as they would do for clinical specimens, and then submit the results to the EQA administrator for performance assessments. The EQA program then analyzes the performance data based on established criteria and returns a summary to each lab, which describes results of how the lab compared with the peers [16,21].

## 2. Ongoing EQA is Critical for SS-Based HIVDR Genotyping

Currently, PT testing for SS-based HIVDR genotyping is offered by several international agencies or programs including the Virology Quality Assurance (VQA) programs funded through the National Institute of Allergy and Infectious Diseases (NIAID) in the U.S.; the HIVDR Typing Proficiency Program through the Quality Control for Molecular Diagnostics (QCMD) in Europe; the Therapeutics, Research, Education, and AIDS Training (TREAT) Asia Quality Assessment Scheme (TAQAS) in Asia; and the Japanese external quality assessment program (JESQ) in Japan [22,23,24,25]. Apart from JESQ which uses lyophilized in-vitro transcribed RNA as panel samples, other programs use clinical samples or viral isolates that have been well-characterized for viral load and DRMs using commercially available kits.

The HIVDR genotyping assay is a complex multi-procedural process involving both sample processing in the lab and subsequent data analysis using sophisticated bioinformatics tools. All available EQA programs allow labs to perform their LDTs as well as the use of commercially available kits such as ViroSeqTM and TRUGENE TM HIV-1 genotyping systems. Sanger sequences are analyzed using specialized software such as RECall or others to create a consensus sequence for each PT specimen. The Stanford HIV Drug Resistance Database or International AIDS Society-USA (IAS-USA) reference DRM list is then used for HIV DRM identification and clinical interpretation [26,27,28]. At present, most laboratories submit the Sanger sequences (in FASTA format), the HIV DRM reports and the specimen information report via web utility. The data is then compiled and assessed against the peers and proficiency scores for each lab are then determined.

Typically, the EQA program would create a consensus sequence for each PT specimen based on the alignment of all submitted sequences derived from it using similar assays (LDTs or specific commercial assay). For instance, the VQA program requires an ≥ 80% absolute nucleotide agreement for the consensus sequence construction [22]. The QCMD from Europe creates a consensus based on the observations from ≥ 60% of the sequences [23], whereas TAQAS from Asia uses a ≥ 70% threshold [24]. Once constructed, the consensus sequences will then be taken as the reference against which the quality of the sequence submissions from individual labs will be assessed. Proficiency scores are calculated generally based on the number of disagreements from the consensus or reference sequence. Labs are also scored on their ability to detect HIV DRMs identified by the majority or reference sequence. Each participant lab then receives an assessment report that includes sequence alignments, homology tables, mutation outputs and total scores [22,23,24,25,29].

## 3. The Development of EQA for NGS-Based HIVDR is Essential

As described above, SS-based HIVDR assays have been widely applied for decades with well-defined EQA strategies in place and many sophisticated EQA programs currently in operation. In contrast, implementation of NGS-based HIVDR testing in clinical HIVDR monitoring is still in its infancy, although such technologies have been broadly applied in the research settings since 2007 [30,31]. Through years of research and development efforts, many NGS HIVDR assay protocols with proven performance have been established for effective sample processing, and some bioinformatics pipelines have also become readily available for general end-users [32,33,34,35]. Besides these LDTs, the Vela Sentosa® SQ HIV-1 Genotyping platform has been approved as the first commercial NGS assay for clinical HIVDR typing by regulatory agencies, including the U.S. Food & Drug Administration. Despite these, the EQA component that helps to ensure consistency and high-quality results is still missing for NGS-based HIVDR assays.

It is acknowledged that several recommendations have been made for standardizing NGS-based clinical tests [2,12,13,14,15,36,37]. However, none of these guidelines are specific for NGS assays targeting viral pathogens with significant intra-host diversity. Some effort had been made to conduct EQA on NGS HIVDR assays using the consensus sequences derived from the NGS reads, to approximate SS outputs, by employing the existing PT panels and EQA strategies originally designed for SS-based HIVDR assays [38]. This approach may serve as a transitional resolution for EQA on NGS HIVDR tests. However, it oversimplifies the complexity and richness of NGS HIVDR data when millions of NGS reads are taken into account in the quantitative assessment. Fully validated EQA strategies specific to NGS-based HIVDR testing remain to be established.

## 4. EQA for NGS-Based HIVDR Assays: Unique Requirements and Challenges

The rapid replication of HIV and the lack of HIV reverse transcription proofreading machinery results in a high mutation rate and highly diversified viral populations or “quasispecies” within a host. Thus, there is a high genetic variability of HIV viruses that already exist among the infected subjects. Consequently, because the HIVDR genotyping assay needs to analyze such a complex, heterogeneous viral population, it will not be as straightforward as sequencing prokaryotic and eukaryotic genomes which have significantly less genetic variation. NGS enables analysis of individual HIV genomes in a quasispecies population with high resolution. However, the high volume and complexity of NGS data further convolute the EQA effort that aims to operationalize NGS HIVDR assays for clinical applications. Some unique requirements and challenges in EQA development for NGS-based HIVDR assays are highlighted below.

### 4.1. PT Panel Design for NGS-Based HIVDR Testing

PT, as part of EQA for NGS-based HIVDR testing, is imperative for the validation of such assays. The panel composition for current SS-based EQA programs is plasma, serum or dried blood spots consisting of donor specimens, clinical isolates or infectious molecular clones [22,23,24,39], as well as lyophilized in-vitro transcribed RNA as panel samples [25]. Similar sample types could be adapted for NGS-based proficiency panels which are well quantitatively characterized for the relative abundance of DRMs found in each sample.

Table 2 summarizes some PT sample options for NGS-based HIVDR assays and their advantages and disadvantages when being utilized for EQA purposes. The use of various infectious molecular clones (IMC) that have defined DRMs could be mixed at different ratios to generate DRMs at different frequencies. The mixed IMC could be accurately characterized by single genome sequencing [40] or by parallel allele-specific sequencing [41]. One of the challenges in using IMC is that spontaneous mutations may be introduced during propagation [42], although such mutations are generally rare and random [43]. The use of plasmid mixtures harboring well-characterized DRMs holds the most promise, where true frequencies of HIV DRMs would be consistent. Although not a true clinical sample, plasmids and plasmid mixtures with confirmed ratios could be established and used as reference materials for detecting DRMs with varied abundance, particularly at lower frequencies, and for monitoring systematic error [32,44]. Alternatively, in vitro transcription of viral RNA from a construct that contains a T7 promoter could be used to generate bulk RNA templates for use as reference materials, similar to those used in the JEQS program [25,45,46,47]. These templates could be spiked into appropriate mediums such as plasma, serum or blood. Theoretically, the transcripts would be clonal with low error due to the T7 RNA polymerase (nucleotide substitution error rate at 10^−4^–10^−5^) [48,49]. The templates could be characterized with NGS and the consensus could be used as the reference material sequence. Deviations from the reference material sequence would be interpreted as RT-PCR/PCR error, PCR bias and sequencing error [3,50].

One of the disadvantages of using IMC, plasmids and in vitro-generated RNA is that these sample types are homogenous viral populations and do not represent authentic clinical specimens that usually contain highly diversified HIV genomes. As a result, the use of such samples oversimplifies the NGS-based HIVDR assay. Plasmids, because they are DNA-based, completely omit the RT-PCR step found in the early steps of the assay. On the other hand, the use of traditional Sanger-based PT samples which better represent clinical specimens are difficult to characterize, and the ground truth of the exact frequencies of the DRMs they harbor may not be reliably determined. Therefore a careful combination of real samples, plasmids, IMC and/or in vitro generated RNAs that contain DRMs of interest and at known abundance may be required to assess the full scope of the NGS-based HIVDR assays.

### 4.2. Data Collection for NGS-Based HIVDR Testing

**Figure 2:**
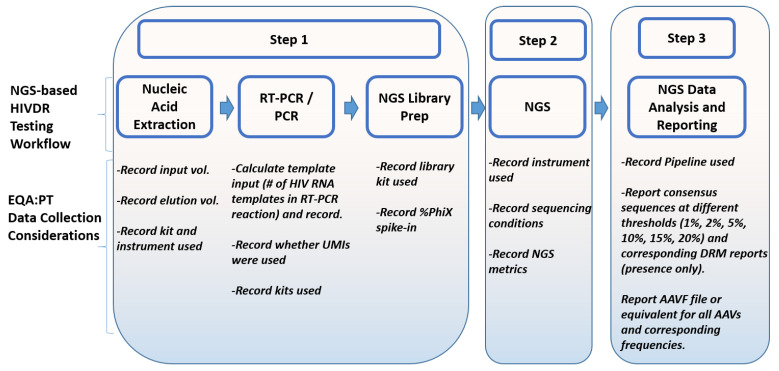
External quality assessment (EQA)/PT for NGS-based HIVDR testing: data collection considerations. (UMI: unique molecular identifier; AAVF: amino acid variant file; AAV: amino acid variant).

The data collected from the participating labs are the only information source for all EQA programs. In SS-based HIVDR EQA programs, laboratories are required to submit the consensus sequences, the HIV DRM reports for the panel specimens and an assay summary or specimen information report. The assay summary includes information about wet-lab methods such as extraction, RT-PCR, PCR, sequencing protocols and software used to analyze sequencing data. While these satisfy all the EQA needs for SS-based HIVDR assays, additional information is required for NGS-based HIVDR assays. Some EQA data collection considerations are summarized in Figure 2.

The recommended data collections aim to facilitate an objective assessment of the lab’s capacity and quality in performing NGS-based HIVDR assay by (1) gauging the initial HIV template input and the subsequent sensitivity for HIV DRM detection it may enable (data from Step 1); (2) evaluating how different NGS platforms may affect the final assay outcome (data from step 2); (3) assessing NGS consensus sequences with specified minor allele identification threshold(s) (data from Step 3); (4) assessing NGS-specific EQA data using quantitative HIVDR reports and comprehensive amino acid variant files (AAVFs) or equivalent, which report all amino acid variants at all frequencies for each specimen (data from Step 3) (Figure 2). Given the ultra-deep sequencing capabilities of NGS platforms, theoretically, NGS-based HIVDR assays could detect all HIV amino acid variations at all frequencies that are above the assay error rate. However, the assay sensitivity limit is ultimately determined by the initial HIV viral input copies, therefore, collecting assay details from Step 1 is essential. The use of unique molecular identifiers (UMIs) is highly recommended when possible, as UMIs can detect the number of actual templates used in the initial RT-PCR reaction and can correct for PCR and sequencing errors [52,53,54].

### 4.3. EQA: Data Assessment and Scoring Strategies for NGS-Based HIVDR Testing

**Figure 3:**
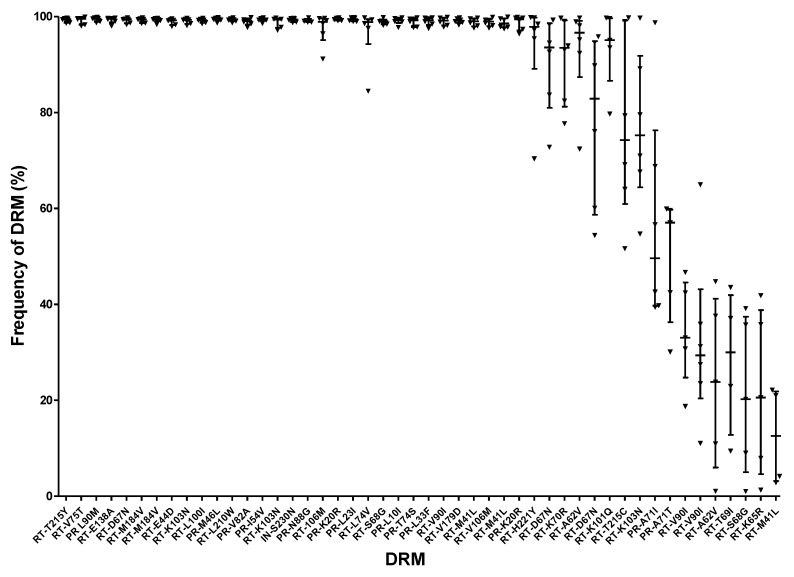
Variation of HIV drug resistance mutations (DRM) frequencies derived from NGS-based HIVDR assays. Six labs received two Virology Quality Assurance (VQA) panels (10 specimens in total) and processed the samples using their own laboratory developed tests (LDT) for NGS-based HIVDR typing. The NGS data (FASTQ files) derived from each of the six labs were analyzed using the HyDRA pipeline [32]. This scatter plot shows the median and interquartile range for HIV DRM frequencies between 1%–100% from each of the six labs. For certain DRMs, some labs did not detect the presence of the DRM and were excluded from the analysis.

In 2018, a joint project involving ten labs in Canada, USA, Mexico and Europe was conducted to assess whether panels from the NIAID’s HIV VQA program could be used for NGS-based proficiency testing [38]. Two panels, each containing five specimens (real donors or plasma spiked with clinical viral isolates), at various viral loads ranging from 3656 to 29,139 copies/ml were distributed to the participant labs and were processed using their independent LDTs for NGS-based HIVDR typing. Consensus sequences derived from all labs using varied NGS platforms were collected at different frequency thresholds specifically, 5%, 10%, 15% and 20% and were compared. Results from the sequence homology analysis which was assessed using the current VQA scoring methods showed high nucleotide concordance at all examined thresholds [38]. To evaluate the DRM frequency readouts, raw NGS reads (FASTQ files) from six of the ten labs were processed through the HyDRA pipeline [32], and the HIV DRM reports derived from the NGS reads were compared (Table S1). Figure 3, which was previously presented at XXVII International HIV Drug Resistance and Treatment Strategies Workshop in Johannesburg, South Africa (October 2018), shows the DRM frequency readouts for all the DRMs found in the two panels at a median threshold of 5%. The frequency of the DRMs widely fluctuates, especially when their abundance was below 90% [55]. The volatility of the DRM frequency results implies that innovative statistical scoring and assessment methods will have to be developed to assess the NGS HIVDR assay outputs from different labs, especially when dealing with the following issues.

#### 4.3.1. Inconsistencies in Detecting DRMs

The data shown in Figure 3 demonstrates that the DRM frequency outputs from each lab participant are inconsistent. In most cases, all labs were able to detect the DRMs present in the VQA samples, albeit at different frequencies. However, there were cases where one or two labs could not detect a DRM, which was otherwise identified by the majority of the labs. One suggestion, raised at the 2019 International Symposium on NGS HIVDR, was that DRM frequency readouts obtained from each lab would not be directly assessed. Instead, the threshold for the NGS consensus sequences should be lowered from 20% to 5%, and the ability of whether the lab could qualitatively detect the presence of the DRM in the group consensus would be assessed. However, even with this method of evaluating NGS data, the challenge of whether a lab receives a penalty for not detecting a mutation at the suggested 5% threshold is complex. Whether the pre-characterization of the panel samples would be considered as the standard, or the standard should base on the consensus of the participants’ results has not been resolved. If the consensus of the participants’ results from PT is set as the standard for evaluating whether a mutation is truly present, the percentage that comprises the consensus is debatable. For example, would the current VQA measure of 80% of participant results comprise the consensus, or would the value of 80% need to be lower to assess MRVs? This method of assessment could use the proposed “assessment panel” described previously by H. Ji et al. (2020) and current EQA scoring strategies could be applied.

#### 4.3.2. Large Variations in DRM Frequencies

One of the main advantages of NGS is that the relative abundance (% frequency) of DRMs can be determined. To assess the accuracy of the detected relative abundance, one would require the proposed “verification panel” with samples for which DRMs and exact frequencies have been well-characterized [56]. However, large variations were observed in DRM frequency readouts from different NGS assays while SS EQA panel specimens were tested. Notably, the higher the median frequencies are, the lower inter-lab variations were observed (Figure 3). If frequency measurement is taken into account, would it be assessed in the frequency intervals (e.g. 5%–20%, 20%–90%, 90%–100%) with different respective deviation thresholds, or would one general deviation be used for assessment for all frequencies? We believe it is fair to apply a higher acceptable deviation cut-off for those DRM frequencies at lower frequency brackets for EQA purposes. The difficulty in determining a method to assess frequency read-outs also highlights the need for more comprehensive studies to better define practical deviation cut-off values for various DRM frequency levels. This type of research would also aide in the development of an appropriate statistical method for DRM frequency assessment and help to identify whether participants can consistently detect MRVs.

#### 4.3.3. Variations in Wet-Lab Methods, NGS Platforms and Bioinformatics Pipelines

Current SS-based EQA strategies for HIVDR genotyping accept LDTs, however, NGS-based HIVDR assays introduce more variations as different NGS technologies and diverse bioinformatics pipelines are utilized [32,33,34,35,57,58,59,60,61,62,63]. NGS platform-specific EQA strategies may not be necessary, although sequence-specific errors for different NGS systems exist [58]. Many different bioinformatics pipelines have been developed for HIVDR testing [32,33,34,35,57,59,60,61,62,63]. Recently, we compared and assessed the performance of five pipelines to detect amino acid variants within specimens and found they were highly concordant. All pipelines demonstrated high sensitivity detecting variants even at a 1% threshold. Specificity, on the other hand, was much better at a 2% threshold versus a 1% threshold. Results from this study imply that a 2% threshold may be a more reliable cut-off for drug resistance mutation calling and reporting when NGS technologies are utilized [64]. As addressed previously, careful documentation of all wet-lab and dry-lab steps may aide in EQA and appropriate remedial recommendations.

## 5. Conclusions

NGS is a powerful tool with the ability to detect variants at low-frequency levels that were otherwise undetectable by Sanger sequencing. Research continues to define the clinical importance of MRVs and inform how drug regimens for HIV treatment should be modified when MRVs for DR are detected. The current EQA strategies for SS-based HIVDR testing are insufficient for NGS-based testing because of the drastically different outputs between the two technologies. Unlike SS, NGS can identify MRVs and provides numerical data on the frequencies of DRMs. Well-characterized panels, including the use of clinical specimens, plasmids and in vitro generated HIV RNA templates, are required to assess the ability of a lab to detect MRVs accurately. Although large variations occur in DRM frequencies when comparing different labs performing NGS-based HIVDR testing on the same panels, continued efforts are being made to develop appropriate assessment methods to address this issue. Future studies to include a larger group to not only assess DRMs but all amino acid variations (AAVs) will provide more information on the comparability between labs and how consistent they are in detecting MRVs. Meanwhile, accommodation of both LDTs and commercial NGS assays, such as the Sentosa® SQ HIV-1 assay, in the perspective EQA programs would be essential. The implementation of EQA for NGS-HIVDR is challenging, but with the urgent need for more accurate assays to detect MRVs and the continued efforts from the field, the thoughts can become a reality.

## Figures and Tables

**Table 1:** Comparison of Sanger and next-generation sequencing (NGS)-based HIV drug resistance (HIVDR) assays.

Item	Sanger Sequencing	NGS
**Extraction**	Required	Required
**RT-PCR**	Required	Required
**PCR**	Required	Required
**Specific sequencing primers**	Multiple specific primers	Not required
**Library preparation**	Not required	Required
**Sequencing reaction**	Single reaction	Massive parallel clonal sequencing
**Data output**	One sequence per sample	Thousands of sequences per sample
**DRM frequency detection threshold**	~20%	~1%
**Qualitative DRM detection**	Enabled	Enabled
**Quantitative DRM detection**	Not applicable	Enabled

**Table 2:** Sample suggestions for proficiency testing (PT) panels for NGS-based HIVDR testing.

Sample Type	Advantages	Disadvantages
Donor Specimens (*plasma, serum, DBS*)	Real specimens	Unpredictable DRMs
	Quasispecies population*	Unknown DRM frequency
		Limited supply
		Complicated and expensive to acquire
Clinical Viral Isolates	Quasispecies population	Unpredictable DRMs
	Known DRMs	Unknown DRM frequency
	Unlimited amount	Viral culture required
	Reusable	Expensive and complicated to prepare
		Minor DRMs may arise during viral culture
Infectious Molecular Clones	Culture of clone-derived isolates	Homogenous population with defined DRMs
	Clone mixtures can be produced	Viral culture required
	Abundant Supply	Minor DRMs may arise during viral culture
	Known DRMs	
	Any DRMs in any genes	
	Any DRM frequency	
Plasmids, Plasmid Mixtures, Synthetic RNA	Known sequences	Homogenous population
	Known DRMs	Plasmids are DNA-based and are not suitable
	Any DRMs in any genes	for RNA related protocol validations
	Any DRM frequency	Plasmids underestimate PCR bias
	Ideal for low-frequency DRMs	
	Ideal for NGS standard	
	Ideal for monitoring systematic error	
	Economical	
	Unlimited amount	
	Stable for storage and transportation	

* Quasispecies refers to a swarm of highly related but genetically different viral variants that arise in a host during replication [51].

## Supplementary Materials

The following are available online at https://www.mdpi.com/1999-4915/12/5/550/s1.
