# Supplementary material for: External Quality Assessment for Next-Generation Sequencing-Based HIV Drug Resistance Testing: Unique Requirements and Challenges
Source: Viruses. 2020 May 16;12(5):550. doi: 10.3390/v12050550 (PMC7291216; doi:10.3390/v12050550)
Supplement: Supplementary file 1 [file viruses-12-00550-s001.pdf]

viruses

MDPI

Supplementary

External Quality Assessment for Next-Generation Sequencing-Based HIV Drug Resistance Testing: Unique Requirements and Challenges

Emma R. Lee [1], Feng Gao [2], Paul Sandstrom [1,3] and Hezhao Ji [1,3,*]

1 National HIV and Retrovirology Laboratories, National Microbiology Laboratory at JC Wilt Infectious Diseases Research Centre, Public Health Agency of Canada, Winnipeg, Manitoba, R3E 3R2, Canada; emmar.lee@canada.ca (E.R.L.); paul.sandstrom@canada.ca (P.S.)

2 Department of Medicine, Duke University Medical Center, Durham, NC, 27710, USA; feng.gao@duke.edu

3 Department of Medical Microbiology and Infectious Diseases, University of Manitoba, Winnipeg, Manitoba, R3E 0J9, Canada

* Correspondence: hezhao.ji@canada.ca; Tel.: 1-204-789-6521

Received: 21 April 2020; Accepted: 14 May 2020; Published: 16 May 2020

Table S1. HIV DRM reports from VQA panels processed NGS-based HIVDR assays and analysed by HyDRA.

Sample Name	Viral Load (c/ml)	Gene	Classification	Surveillance	Mutation	Lab 1	Lab 2	Lab 3	Lab 4	Lab 5	Lab 6	Average	Median
24.1	7,815	PR	Other	No	V82I	.	1.23	.	.	.	.	1.23	1.23
		RT	NRTI	Yes	M184V	.	1.28	.	.	.	.	1.28	1.28
		RT	NRTI	Yes	T215C	69.16	79.36	51.65	64.04	99.01	99.76	77.16	74.26
		RT	NRTI	Yes	T215D	2.11	3.24	.	10.71	.	.	5.35	3.24
		RT	NRTI	Yes	T215S	0.07	0.2	5.86	0.03	0.04	0.01	1.04	0.055
		RT	NRTI	Yes	T215Y	5.26	4.42	.	6.79	.	.	5.49	5.26
		RT	NNRTI	Yes	P225H	.	1.24	.		.	.	1.24	1.24
		IN	Accessory	No	E157Q	.	.	.	3.7		.	3.7	3.7
		IN	Other	No	S230N	99.26	98.91	99.61	99.64	98.77	99.67	99.31	99.44
24.2	18,023	PR	Other	No	K20R	96.43	97.4	99.73	99.74	99.19	99.91	98.73	99.46
		RT	NRTI	Yes	D67E	.	0.54	.	.	1.33	.	0.935	0.935
		RT	Other	No	S68G		1.01				.	1.01	1.01
		RT	NNRTI	Yes	P225H	.	1.11	.	.	0.65	.	0.88	0.88
		IN	Major	Yes	Q148K	.	.	.	.	1.06	.	1.06	1.06
		IN	Accessory	No	G163R	99.19	99.25	99.69	99.54	98.77	99.63	99.35	99.4
24.3	26,372	RT	NRTI	Yes	M41L	97.86	97.89	99.31	99.58	97.49	99.92	98.68	98.6
		RT	NRTI	Yes	K65R	.	1.14	.	.	.	.	1.14	1.14
		RT	Other	No	S68G	99.1	98.24	99.02	99.08	98.45	99.83	98.95	99.05
		RT	NRTI	Yes	L74I	19.19	5.54	3.74	.	0.58	.	7.26	4.64
		RT	NRTI	Yes	V75T	99.64	98.09	99.49	99.65	98.35	99.94	99.19	99.57
		RT	Other	No	V90I	98.91	97.84	99.46	99.73	98.22	99.88	99.01	99.19
		RT	Other	No	K103R	4.17	6.34	3.07	.	.	1.19	3.69	3.62
		RT	NNRTI	Yes	V106M	98.67	98.16	99.31	99.26	97.66	99.91	98.83	98.97
		RT	NNRTI	No	V179D	98.82	98.29	99.3	99.61	99.02	99.78	99.14	99.16
		RT	NRTI	Yes	M184V	23.95	11.02	3.74	.	.	.	12.9	11.02
24.4	29,139	RT	NRTI	Yes	M41L	98.65	97.48	not done	99.46	98.57	99.7	98.77	98.65
		RT	NRTI	Yes	D67E	.	1.2		.	1.54	.	1.37	1.37
		RT	Other	No	T69N	.	0.84		.	1.31	.	1.08	1.08
		RT	NNRTI	Yes	K103N	84.59	80.76		84.93	97.88	99.49	89.53	84.93
		RT	NNRTI	Yes	Y181C	2.44	3.45		7.27	.	.	4.39	3.45

		RT	NRTI	Yes	M184V	96.78	95.59		91.74	73.81	82.38	88.06	91.74
		RT	NRTI	Yes	T215Y	99.36	98.54		99.63	98.64	99.49	99.13	99.36
		RT	Other	No	K238R	.	0.73		.	0.66	18.8	6.73	0.73
		PR	Other	No	L10I	98.96	98.56	99.36	99.56		99.66	99.22	99.36
		PR	Accessory	Yes	L23I	99.17	98.76	99.64	99.77		99.91	99.45	99.64
		PR	Accessory	No	L33F	99.11	98.19	99.59	99.59		99.86	99.27	99.59
		PR	Major	Yes	M46L	99.31	99.07	92.82	99.58		99.64	98.08	99.31
		PR	Major	Yes	I54V	99.21	98.4	99.29	99.5		99.7	99.22	99.29
		PR	Accessory	No	Q58E	.	.	10.92	2.25		.	6.59	6.585
		PR	Other	No	A71I	62.36	33.63	44.64	60.98		96.24	59.57	60.98
		PR	Other	No	A71T	36.74	65.23	54.84	38.71		.	48.88	46.78
		PR	Major	Yes	V82A	99.21	98.58	99.51	97.32		99.74	98.87	99.21
		PR	Major	No	N88G	99.36	98.38	99.27	99.23		99.68	99.18	99.27
		PR	Major	Yes	L90M	99.54	98.83	99.63	99.78		99.76	99.51	99.63
24.5	6,424	RT	NRTI	Yes	M41L	98.14	97.85	99.34	99.55	not done	81.54	95.28	98.14
		RT	Other	No	M41I	.	0.1	0.01	0.02		18.18	4.58	0.06
		RT	NRTI	No	E44D	99.01	97.71	98.98	99.41		99.4	98.9	99.01
		RT	NRTI	No	A62V	98.49	44.96	86.6	92.34		99.74	84.43	92.34
		RT	NRTI	Yes	D67N	99.31	98.51	99.37	99.38		99.75	99.26	99.37
		RT	NRTI	Yes	L74I	5.54	12.34	.	2.69		.	6.86	5.54
		RT	NRTI	Yes	L74V	93.58	85.36	98.8	96.51		99.82	94.81	96.51
		RT	NNRTI	Yes	L100I	99.2	98.2	99.56	99.8		99.12	99.18	99.2
		RT	NNRTI	Yes	K103N	99.21	97.66	98.99	99.11		99.66	98.93	99.11
		RT	NRTI	Yes	L210W	99.11	98.95	99.51	99.23		99.87	99.33	99.23
		RT	NRTI	Yes	T215Y	99.25	98.62	99.59	99.63		99.46	99.31	99.46
		RT	NNRTI	No	H221Y	98.74	98.81	88.25	95.72		99.84	96.27	98.74
		PR	Other	No	A71T						1.3	1.3	1.3
		PR	Other	No	T74S	98.99	97.86	99.51	99.51	97.76	99.79	98.9	99.25
		RT	NRTI	Yes	M41L	2.86	4.14	21	.	.	22.14	12.535	12.57
		RT	NRTI	Yes	K65R	.	1.08	.	.	.	.	1.08	1.08
26.1	16,685	RT	NRTI	Yes	D67G	.	1.82	.	.	.	.	1.82	1.82
		RT	NRTI	Yes	D67N	94.51	92.64	83.72	99.3	98.36	72.79	90.22	93.58
		RT	NRTI	Yes	K70R	93.92	93.06	82.42	99.71	99.08	77.68	90.98	93.49
		RT	Other	No	V90I	23.52	27.49	35.89	64.97	11.03	31.24	32.36	29.37
		RT	NNRTI	No	V108I	.	1.85	.	.	.	.	1.85	1.85

		RT	NRTI	Yes	M184V	99.41	98.38	99.44	99.62	99.22	99.68	99.29	99.43
		RT	NRTI	Yes	T215F	.	7.6	19.03	.	.	31.68	19.44	19.03
		RT	NRTI	Yes	T215Y	9.15	5.16	4.75	.	.	.	6.35	5.16
		RT	NRTI	Yes	K219E	.	1.02	.	.	.	.	1.02	1.02
		RT	NNRTI	Yes	P225H	.	1.63	.	.	.	.	1.63	1.63
		PR	Other	No	L10I	99	97.76	99.46	99.64	98.97	99.79	99.1	99.23
		PR	Accessory	Yes	L23I	99.19	99	99.7	99.75	99	99.86	99.42	99.45
		PR	Accessory	No	L33F	98.98	97.55	99.56	99.7	98.49	99.77	99.01	99.27
		PR	Major	Yes	M46L	99.31	98.71	99.47	99.67	99.55	99.83	99.42	99.51
		PR	Major	Yes	I54V	99.29	98.61	99.31	99.64	99.3	99.79	99.32	99.31
		PR	Accessory	No	Q58E	3.67	.	.	12.6	.		8.14	8.14
		PR	Other	No	A71I	56.66	68.8	39.76	42.59	98.76	39.42	57.66	49.63
		PR	Other	No	A71T	42.48	30.09	59.89	57.03	.	59.6	49.82	57.03
		PR	Major	Yes	V82A	99.3	98.54	99.34	97.75	98.9	99.78	98.94	99.1
		PR	Major	No	N88G	99.24	99.06	98.86	99.21	98.87	99.88	99.19	99.14
		PR	Major	Yes	L90M	99.57	98.74	99.63	99.75	99.01	99.56	99.38	99.57
		RT	NRTI	Yes	M41L	98.73	98.09	99.31	99.71	97.64	99.18	98.78	98.96
26.2	4,513	RT	NRTI	No	E44D	99.36	98.34	99.27	99.57	97.97	99.7	99.04	99.32
		RT	NRTI	No	A62V	92.39	98.13	72.41	95.2	98.94	.	91.41	95.2
		RT	NRTI	Yes	K65R	.	.	.	.	1.46	.	1.46	1.46
		RT	NRTI	Yes	D67N	99.44	98.71	99.53	99.46	98.36	99.79	99.22	99.45
		RT	Other	No	T69N	.	.	.	.	1.92	.	1.92	1.92
		RT	NRTI	Yes	L74I	.	.	14.28	.	.	.	14.28	14.28
		RT	NRTI	Yes	L74V	99.18	97.58	84.48	99.36	98.58	99.76	96.49	98.88
		RT	NNRTI	Yes	L100I	99.31	98.54	99.49	99.69	98.78	99.26	99.18	99.29
		RT	NNRTI	Yes	K103N	99.35	98.14	99.28	99.46	98.59	99.69	99.09	99.32
		RT	NRTI	Yes	L210W	99.3	98.73	99.38	99.56	99.1	99.79	99.31	99.34
		RT	NRTI	Yes	T215Y	99.65	98.65	99.5	99.7	98.75	99.42	99.28	99.46
		RT	NNRTI	No	H221Y	95.39	98.46	70.37	99.76	97.27	99.84	93.52	97.87
		IN	Major	Yes	Q148K			.	.	1.22	.	1.22	1.22
		IN	Other	No	S230N	.	.	.	6.68	.	.	6.68	6.68
		PR	Other	No	K20R	99.21	99.39	99.75	99.67	99.15	99.44	99.44	99.42
26.3	18,213	RT	NRTI	Yes	M41L	17.6	2.11	.	.	.	.	9.86	9.86
		RT	NRTI	No	A62V	10.93	37.58	23.81	44.76	.	1.04	23.62	23.81
		RT	NRTI	Yes	K65R	7.92	35.82	20.57	41.84	.	1.29	21.49	20.57

		RT	NRTI	Yes	D67N	89.76	60.1	76.07	54.39	95.85	94.6	78.46	82.92
		RT	Other	No	S68G	9.01	35.73	20.24	39.14	.	1.01	21.03	20.24
		RT	Other	No	T69I	9.42	37.11	22.92	43.54	.	.	28.25	30.02
		RT	NRTI	Yes	V75T	8.99	35.21	28.3	37.84	.	.	27.59	31.76
		RT	Other	No	V90I	14.35	3.07	.	3.85	95.14	.	29.1	9.1
		RT	NNRTI	No	L100V	.	.	.	.	1.44	.	1.44	1.44
		RT	Other	No	K101Q	79.72	95.09	99.64	93.54	.	99.74	93.55	95.09
		RT	NNRTI	Yes	K103N	99.28	97.83	99.49	99.74	97.18	99.49	98.84	99.39
		RT	NNRTI	Yes	V106M	99.22	98.57	91.19	99.66	96.44	99.69	97.46	98.9
		RT	Other	No	V106I	.	.	8.5	.	.	.	8.5	8.5
		RT	NNRTI	No	E138A	99.45	99.03	99.69	99.73	98.06	99.63	99.27	99.54
		RT	NRTI	Yes	M184V	99.41	98.61	99.58	99.75	98.69	99.46	99.25	99.44
26.4	6,506	RT	NRTI	Yes	D67G	.	1.58	.	.	not done	.	1.58	1.58
		RT	NRTI	Yes	F77L	.	1.04	.	.		.	1.04	1.04
		RT	NNRTI	Yes	G190E	5.71	.	.	.		.	5.71	5.71
		IN	Major	Yes	F121Y	.	7.65	.	.		.	7.65	7.65
26.5	3,656	PR	Major	Yes	I47V	.	1.02	.	.	.	.	1.02	1.02
		PR	Other	No	V11I	.	1.38	.	.	.	.	1.38	1.38
		RT	Other	No	V90I	42.43	30.74	18.73	33.04	46.71	.	34.33	33.04
		RT	NNRTI	Yes	K103N	67.66	79.53	89.19	70.98	54.72	99.72	76.97	75.26
		IN	Accessory	No	T97A	1.41	.	.	.	.	.	1.41	1.41
		IN	Accessory	No	S153F	.	.	.	1.91	.	.	1.91	1.91

Two panels from the National Institute of Allergy and Infectious Diseases (NIAID) VQA program, each consisting of five samples, were processed by six labs using their own LDT for NGS-based HIVDR assays. A total of three samples from two labs were not processed. The NGS data (FASTQ files) were analysed using the bioinformatic pipeline HyDRA. The portions highlighted in grey indicate the DRMs not identified by all six participating labs.
